# A Nearly Lethal Screw: An Unusual Cause of Recurrent Bradycardia and Asystole Episodes after Fixation of the Cervical Spine

**DOI:** 10.1155/2017/3748930

**Published:** 2017-10-12

**Authors:** Amit Frenkel, Yair Binyamin, Evgeni Brotfain, Leonid Koyfman, Aviel Roy-Shapira, Ilan Shelef, Moti Klein

**Affiliations:** ^1^General Intensive Care Unit, Soroka University Medical Center and the Faculty of Health Sciences, Ben-Gurion University of the Negev, Beer-Sheva, Israel; ^2^Department of Anesthesiology, Soroka University Medical Center and the Faculty of Health Sciences, Ben-Gurion University of the Negev, Beer-Sheva, Israel; ^3^Department of General Surgery, Trauma Unit, Soroka University Medical Center and the Faculty of Health Sciences, Ben-Gurion University of the Negev, Beer-Sheva, Israel; ^4^Department of Radiology, Soroka University Medical Center and the Faculty of Health Sciences, Ben-Gurion University of the Negev, Beer-Sheva, Israel

## Abstract

We present a case of a 51-year-old man who was injured in a bicycle accident. His main injury was an unstable fracture of the cervical and thoracic vertebral column. Several hours after his arrival to the hospital the patient underwent open reduction and internal fixation (ORIF) of the cervical and thoracic spine. The patient was hospitalized in our critical care unit for 99 days. During this time patient had several episodes of severe bradycardia and asystole; some were short with spontaneous return to sinus and some required pharmacological treatment and even Cardiopulmonary Resuscitation (CPR). Initially, these episodes were attributed to the high cervical spine injury, but, later on, CT scan suggested that a fixation screw abutted on the esophagus and activated the vagus nerve by direct pressure. After repositioning of the cervical fixation, the bradycardia and asystole episodes were no longer observed and the patient was released to a rehabilitation ward. This case is presented in order to alert practitioners to the possibility that, after operative fixation of cervical spine injuries, recurrent episodes of bradyarrhythmia can be caused by incorrect placement of the fixation screws and might be confused with the natural history of the high cervical cord injury.

## 1. Introduction

Spinal cord injuries are most often caused by physical trauma. It is estimated that in the USA the incidence of spinal cord injury is about 40 cases per 1 million people per year, which amounts to 12,000 annually [1].

The most common cause is motor vehicle accidents, followed by falls, violence such as gunshot wounds, and then sports injuries [2].

Severe SCI usually lead to either temporary or permanent functional dysfunction [3]. This dysfunction translates into loss of muscle tone, sensation, or autonomic function in body organs served by the spinal cord below the level of the lesion. Injuries can occur at any level of the spinal cord and can be classified as “complete injuries,” with total loss of sensation and muscle tone, or “incomplete injuries,” if some of these functions are preserved [4].

In high cervical spine injury, particularly cephalad to C5, recurrent episodes of bradycardia and hypotension sometimes occur [5]. This autonomic dysfunction is caused by loss of sympathetic innervation and may last from several hours to several weeks [6] and is sometimes referred to as “spinal shock.” The later term refers to the paradoxical response of the lower motor neuron to loss of inhibitory upper motor neuron signal. This phenomenon should not be confused with “neurogenic shock” which refers to the hemodynamic effects of high spinal shock transection.

In SCI surgical intervention may be necessary to stabilize the spine, to restore the alignment of the vertebra, or to relief direct pressure from the cord. While timing of surgery is still under debate [7], some studies have shown that early surgical intervention (within 24 hours of injury) is associated with better outcomes [8].

Like any other operation, cervical spine surgery carry a risk of complications, which range from nonspecific such as infection and bleeding, to complications related to anesthesia and technical errors. These complications may manifest as wound infection, discitis, and dural tears with cerebrospinal fluid leak [9]. Anterior technique for cervical spine fixation carries some unique complications; among them are recurrent or superior laryngeal nerve injuries, hypoglossal nerve injuries, esophageal trauma, or damage to the vertebral and carotid artery [10].

Hemodynamic disturbances can be anticipated during and after operations on the spine and can be caused by prone positioning, bleeding, and neurogenic deficit. However, in addition to bleeding, cardiovascular emergencies may be related to stimulation of specific nerve roots or autonomic dysfunction or be rarely due to venous air embolism and often require intensive resuscitative efforts. The majority of these adverse events are reported in either cervical or upper thoracic spine surgeries [11]. In the case presented, the compression of the screw used for fixation led to vagus nerve stimulation and was the cause for the bradycardia events rather than the high spinal cord injury itself. The purpose of the presentation is to alert physicians to this possibility.

## 2. Case Presentation

A previously healthy 51-year-old man was admitted to our critical care unit after a bicycle accident. On arrival, the patient was hemodynamically stable but sedated and on mechanical ventilation.

Before intubation, at the scene, he had flaccid paralysis of both legs and left arm, with minimal movements of the right arm.

CT demonstrated bilateral small pneumothoraxes, bilateral pulmonary contusions, and dislocated fractures of the vertebral spine at C4-C5.

An MRI demonstrated a significant narrowing of the cervical spinal canal with myelomalacia signs, edema, and hemorrhagic contusion at the level of C3–C6. The spinal cord was also compressed at the level of D5 due to vertebral fractures at D7.

An emergency cervical discectomy of C4-5 followed by fusion of D2-3 and D6–8 was performed and the patient was transferred to the ICU.

During his ICU stay the patient developed several events of extreme bradycardia which occasionally deteriorated to asystole.

Some of these events resolved spontaneously within a few seconds, while some events required pharmacological treatment or CPR.

Initially, these events were attributed to neurological disturbances which have been well described in high spinal cord injuries [5] and it was hoped that they will either resolve with time or if not, a pacemaker will be required.

After a few weeks of observation the nursing staff reported that these events always occurred when the patient was in the left lateral decubitus position. This observation suggested an anatomical problem.

**Figure 1:**
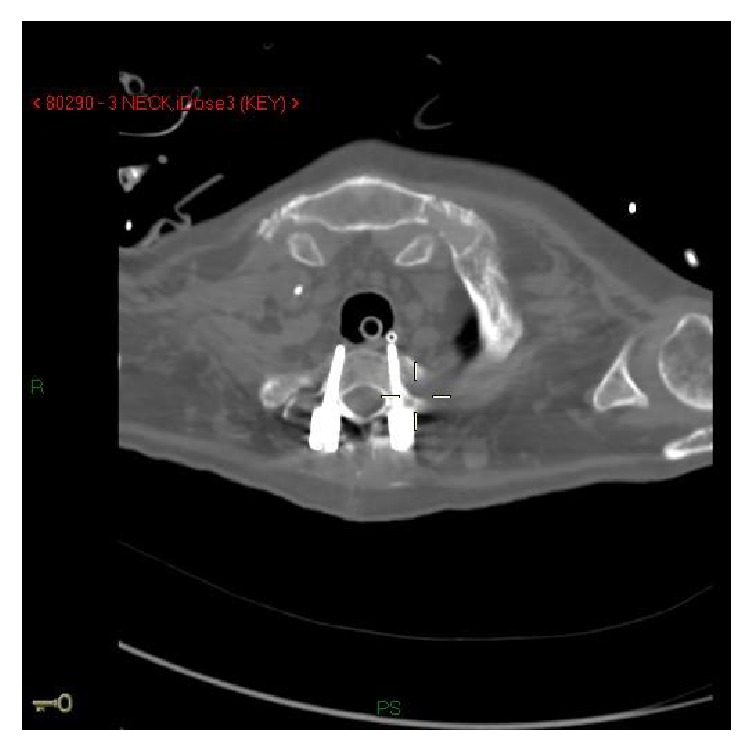
Computed tomography (axial view): fixation screw abetting the esophagus.

A second CT scan demonstrated that one of the screws used in the process of ORIF at the level of C5 abutted on the esophagus and was compressing the vagus nerve when the patient was in the left lateral decubitus position (Figure 1).

Following these findings, the patient was taken to the operating theater and the offending screw was repositioned. The postoperative course was uneventful: soon after surgery, the temporary pacemaker placed before the operation was removed and the patient was transferred to a rehabilitation ward. Over three years of follow-up no episodes of dysrhythmia occurred.

## 3. Discussion

After high cervical cord disruption dysrhythmias are commonly seen due to disturbance in autonomic response and are to be expected [5].

In this case the dysrhythmias episodes were caused by a misplaced screw which abutted on the vagus nerve and compressed the vagus when the patient was in the left decubitus position.

We have not been able to find a prior description of a similar case of severe bradycardia and asystole caused by iatrogenic compression of the vagus nerve in the literature. However, these are possible complications of cervical spine surgery and should be taken into consideration in the differential diagnosis of paroxysmal bradycardia and asystole in patients undergoing similar procedures.
